# A pilot study of the utility of a hospital-based school program for pediatric patients with cardiac diagnoses

**DOI:** 10.3389/fped.2024.1502378

**Published:** 2024-12-11

**Authors:** Lia K. Thibodaux, Ashley L. Orr, Debra L. Reisinger, Jill Fodstad, Guang Xu, Kristin Wikel, Michelle Curtin

**Affiliations:** ^1^ Department of Neurology, Wake Forest School of Medicine, Winston-Salem, NC, United States; ^2^ Marian University College of Osteopathic Medicine, Indianapolis, IN, United States; ^3^ Department of Pediatrics, University of Cincinnati College of Medicine, Cincinnati, OH, United States; ^4^ Division of Behavioral Medicine and Clinical Psychology, Cincinnati Children’s Hospital Medical Center, Cincinnati, OH, United States; ^5^ Department of Psychiatry, Indiana University School of Medicine, Indianapolis, IN, United States; ^6^ Department of Special Education, Ball State University Teacher’s College, Muncie, IN, United States; ^7^ Department of Pediatrics, Wake Forest School of Medicine, Winston-Salem, NC, United States

**Keywords:** school, academics, development, education, cardiology

## Abstract

**Introduction:**

Pediatric patients with complex cardiac diagnoses are at increased risk for physical, cognitive, and developmental complications. Formalized school support [i.e., individualized education programs (IEPs), Section 504 Accommodation Plans (Section 504 Plans)] that addresses the needs of these patients is necessary, and hospital-based school programs (HBSPs) have the potential to bolster the acquisition of academic support. In this pilot study, we look at the impact of one such HBSP.

**Methods:**

Retrospective demographic and school support data for pediatric cardiac patients were analyzed.

**Results:**

Our sample included 29 pediatric cardiac patients spanning two academic years. These patients had 100 HBSP encounters and 82 inpatient and 12 outpatient encounters, with 68.9% of patients having multiple encounters in a single year and 44.8% of patients being seen in both years. The HBSP made recommendations for patients to receive IEPs (*N* = 8) and Section 504 Plans (*N* = 13). The patients also submitted requests for medical homebound(*N* = 27), obtaining releases of information (*N* = 39), submitting medical reports (*N* = 10), and completing certificates of incapacity (*N* = 7). Statistical analyses revealed no significant relationships with patients entering or receiving a recommendation for an IEP or Section 504 Plan in any of their encounters with the HBSP on the basis of sex, race/ethnicity, school level, or rates of encounters in this sample.

**Discussion:**

Similar to previous studies, these patients had high rates of IEPs/Section 504 Plans in place and continued to receive school recommendations through the HBSP. A high use of the HBSP was seen in the total number of encounters and communications (i.e., submitting to the school of record requests for classroom placement changes via medical homebound). Working with the HBSP provided access to information, formal support recommendations, and communication between medical and school settings in the form of changes in school status.

## Introduction

Long-term outcomes with regard to neurocognitive and developmental functioning are variable for children with congenital or acquired cardiac conditions (1–4). However, information on the impact of cardiac conditions on long-term educational achievement and adult quality of life is limited (5, 6). With increasing life expectancy for pediatric patients with cardiac conditions, this is an area of growing importance (7, 8). Patients with congenital or acquired cardiac conditions are well-known to have weaknesses in cognitive skills, learning/academic skills, attention, and executive function in childhood (9–11). Despite what is known about these challenges, there is a lack of literature delineating specific recommendations regarding beneficial developmental, cognitive, and academic supports in the school environment, although children with cardiac diagnoses show higher rates of utilization of special education services (10–12). The 2012 Scientific Statement from the American Heart Association, which includes guidance on surveillance, screening, and evaluation for developmental disorders in patients with congenital heart disease (CHD), encourages medical collaboration with educators (13), but specific services, the role of the pediatric cardiologist in obtaining services, and the processes for doing so are not yet available. Hospital-based school programs (HBSPs) are one potential form of support to address the multifaceted needs impacting school performance and outcomes of pediatric patients with cardiac diagnoses.

HBSPs employ trained teachers to support patients, families, and the medical team in the area of academic and school needs, promoting positive school experiences and facilitating discussion between stakeholders (14). In coordination with ongoing medical care, HBSPs provide inpatient academic and learning support to address the varying needs of pediatric patients and have been used to bridge the academic gap from the stage of hospital inpatient care to eventual reintegration into school (15).

In considering the unique school needs of individuals with cardiac diagnoses, our team examined the school supports and recommendations for children engaging with the HBSP at Riley Hospital for Children, a large midwestern hospital. Specifically, we describe a pilot sample of the engagement of pediatric cardiology patients with the HBSP in their acquisition of academic support for school reintegration after hospitalization. To our knowledge, this is the first paper to explore the delivery of services by an HBSP for pediatric patients with complex cardiac conditions.

## Materials and methods

### Program and services

Described by Rodríguez et al., the HBSP in this manuscript is part of the Riley Hospital for Children, a midwestern children's hospital with 354 inpatient beds (15). Key academic, school and HBSP terms used throughout are defined and described further in Table 1. This HBSP provides a number of services to inpatients, including direct instruction, administration of state-mandated assessments, assistance in school reintegration as part of hospital discharge planning, and support to the patient and their family for any identified school needs with the patient's school of record (15). HBSP reintegration services include proposing and supporting the development of formal supports within the school setting, such as Section 504 Accommodation Plans (Section 504 Plans) or individualized education programs (IEPs). The structure, funding, and services provided by HBSPs in the United States are diverse (14). This HBSP was funded through donations and the Indiana Department of Education (DoE).

**Table 1:** Common terms in education.

Term	Definition
Certificate of incapacity (16)	A form that describes a patient's illness or condition that prevents their attendance at school; varies by state.
Direct instruction (17)	A traditional model of education in which the content to be learned is presented by a teacher directly to the learner; this includes structured lessons, teaching activities, and constant student evaluation.
General education (18, 19)	Standard, state-determined public school curriculum that individuals receive when matriculating in the school systems.
IEP (18, 19)	A written legal document, tailored to a child with a qualifying disability that is developed, reviewed, and revised annually. This includes a statement of present academic achievement, functional performance, measurable annual goals, and special education and related services used.
Medical homebound (19)	Home services provided to students who are unable to attend school due to their medical condition.
Medical report form	A document used by medical professionals that contains information about a patient's medical treatment as pertinent to the school setting.
Section 504 Plan (20, 21)	This plan grants students with disabilities support to access the state-determined curriculum and meet standardized learning expectations in a general classroom.
Special education or exceptional children's programming (18, 19)	Instruction designed to meet the needs of a student with a disability as defined in the IEP, including classroom placement, curriculum expectations, and access to developmental, behavioral, academic, and social supports, among others.

### Participants

A total of 69 pediatric cardiac patients had encounters with the HBSP during a 2-year period. From this group of patients, those with non-primary cardiac illnesses (e.g., infectious etiology) or disorders that could not be determined to be of primary cardiac cause (e.g., arrest) were excluded. The final group included in this study was made up of 29 patients seen in the cardiology service, those who had diagnoses of hypoplastic left heart syndrome (HLHS) and those who underwent a precardiac transplant or postcardiac transplant. Table 2 provides demographic information on these patients. Of the 29 patients, there were 7 patients with an HLHS (24.1%), 14 patients who underwent a precardiac transplant (48.3%), 7 patients who underwent a postcardiac transplant (24.1%), and 1 patient who had an HLHS and underwent a cardiac transplant (3.4%). The mean age of the patients seen during the first encounter across the two academic years was 11.28 (SD = 4.01), and the age of the patients ranged from 6 to 17 years.

**Table 2:** Participant demographics.

Demographic	% (*N*)
Sex	
Male subjects	41.38% (12)
Female subjects	58.62% (17)
Race	
White	72.41% (21)
Black	10.34% (3)
Other	6.90% (2)
More than one	6.90% (2)
American Indian or Alaska Native	3.45% (1)
Ethnicity	
Hispanic or Latino	20.69% (6)
Non-Hispanic or Latino	79.31% (23)

### Consent and IRB approval

Engagement with the HBSP required written consent from the legal guardians of the patients. All consent records were stored in accordance with The Family Educational Rights and Privacy Act (22) in accordance with federal education law and also the Health Insurance Portability and Accountability Act (23). The HBSP liaisons communicated with the inpatient medical team via the electronic medical record, team rounds, and consultations. This study was IRB-approved by the Indiana University School of Medicine.

### Data collection

Recruitment of patients was done through standard HBSP processes during inpatient hospitalization. Any inpatient deemed medically stable by the primary team was offered school support by the HBSP on the first school day of the hospitalization. Data were collected during the 2021–2022 and 2022–2023 school years, following up patients longitudinally during this period while they received services from the HBSP. Information regarding HBSP engagement prior to the study period was not available, and as such, no estimates were available about prior interactions and impact.

HBSP teachers entered state DoE–required data after each patient or associated school interaction into a protected REDCap (24, 25) database hosted by the hospital system. Information on patients with cardiac diagnoses was extracted from the complete HBSP database. Variables included patient demographics, school information, patient-specific school information (e.g., level, presence of educational services), medical care factors, and HBSP service provision. The documented HBSP services included recommendations made for the initiation of or change in an existing IEP or Section 504 Plan and when the recommendation was made. In addition, the completion of different forms impacting school needs was tracked by the HBSP, including a release of academic information, medical homebound, medical report form, or certificate of incapacity. Furthermore, school meeting information was documented, including meeting frequency, purpose, excuse from standardized testing, and peer education offerings. Finally, the date HBSP services ended, the indication, and any follow-up consultation meetings after discharge were recorded by HSBP teachers.

### Data analysis

Analyses were conducted in Microsoft Excel and SPSS version 27 (26). Demographic descriptive statistics such as patient age, sex, race, and ethnicity were calculated using the first encounter. Because of the categorical nature of the data, frequency counts and percentages were used to describe the sample. Fisher's exact tests were conducted to analyze the relationship between school support and patient/school demographic information.

## Results

### Patient and encounter descriptives

Across the two academic years, 29 patients with cardiac conditions received HBSP services. Seven patients enrolled in the 2021–2022 academic year, 9 patients in the 2022–2023 year, and 13 (44.83%) in both academic years. Table 2 describes the patient demographics for sex and race/ethnicity. Twenty of the 29 (68.97%) patients had multiple encounters in a single year. The average age of the patients was 11.28 years (range: 6–17 years), with 14 patients being 6–10 years of age, 10 patients 11–15 years, and five patients 16–20 years old. This group had 100 encounters with the HBSP, wherein 82 of these encounters were for inpatient stays and 12 took place during outpatient treatment. The average number of repeat encounters for patients seen by the HBSP was 3.45 (SD = 2.79). The length of stay for those seen in their inpatient unit averaged 24.35 days (SD = 34.55) with a range of 1–267 days across all encounters.

Across both academic years (*N* = 29), the majority of patients attended traditional public school (75.86%), were elementary school students (51.72%), and were receiving their academic services in a general education classroom (89.65%) during their first encounter with the HBSP (Table 3). Eleven patients (37.93%) had an IEP in place prior to their first encounter. The HBSP made a total of eight recommendations for patients to receive an IEP during their encounters. Six additional patients acquired an IEP during this period, and three of these patients were recommended for an IEP by the HBSP. By the end of HBSP engagement, 17 patients had an IEP (58.62%). In addition, there were four (13.79%) patients who had an existing Section 504 Plan in place prior to their first encounter with the HBSP. The HBSP made 13 recommendations for patients to receive a Section 504 Plan. One patient acquired a Section 504 Plan with a recommendation from the HBSP, resulting in five patients with a Section 504 Plan in the cohort (17.24%).

**Table 3:** Participant school characteristics at first encounter with the HBSP.

Characteristic	Percentage (*n*)		
	All academic years (*n* = 29)	2021–2022 (*n* = 20)	2022–2023 (*n* = 9)
School type			
Public	75.86% (22)	75.00% (15)	77.78% (7)
Charter	6.90% (2)	5.00% (1)	11.11% (1)
Private	6.90% (2)	5.00% (1)	11.11% (1)
Virtual only	10.34% (3)	15.00% (3)	—
School level			
Elementary	51.72% (15)	50.00% (10)	55.56% (5)
Middle/intermediate	13.79% (4)	20.00% (4)	—
High	34.48% (10)	30.00% (6)	44.44% (4)
Classroom type			
General education	89.66% (26)	85.00% (17)	100.00% (9)
Medical homebound	3.45% (1)	5.00% (1)	—
Outside placement	6.90% (2)	10.00% (2)	—

The HBSP also communicated with schools by submitting several types of materials across all encounters. This included obtaining a release of information with the school for bidirectional communication (*N* = 39) and submitting the following: medical homebound recommendations (*N* = 27), medical report forms (*N* = 10), and certificates of incapacity (*N* = 7). See Table 1 for additional information on the various support recommendations. Finally, the HBSP team provided an average of 13.13 school consultations (SD = 22.87; range: 1–143) and 9.83 direct instruction sessions (SD = 25.21; range: 0–175) to patients across all encounters.

### Fisher’s exact test analysis for school and demographic variables

Fisher's exact tests revealed no significant relationship between race (White vs. non-White) and entering the HBSP with an IEP or Section 504 Plan or receiving a recommendation for an IEP or Section 504 Plan during any of their encounters with the HBSP *(p*s > 0.092)*.* Similarly, there was no significant relationship between sex and entering the HBSP with an IEP or Section 504 Plan or receiving an IEP or Section 504 Plan recommendation during or after any of their HBSP encounters *(p*s > 0.264)*.* Finally, no significant relationship was found between ethnicity (Hispanic vs. non-Hispanic) and entering the HBSP with an IEP or 504 Plan or receiving an IEP or Section 504 Plan recommendation during or after any of their HBSP encounters *(p*s > 0.058).

Fisher’s exact test was performed to examine the relationship between the patient's school level (elementary, middle/intermediate, and high school) and whether they entered HBSP services with an IEP or Section 504 Plan. This relationship was not significant (*p* = 0.061)*.* Similarly, when examining the school level and IEP or Section 504 Plan recommendations during or after their HBSP encounters, no significant relationship emerged (*p* = 0.429).

Using the mean number of encounters to divide the group (*M* = 3.45), we divided patients into those who had three or fewer encounters or four or more encounters with the HBSP. Fisher's exact tests revealed no significant relationship between rates of encounters and entering HBSP services with an IEP or Section 504 Plan in place (*p* = 0.060). Similarly, no significant relationship was found between rates of encounters and receiving a recommendation for an IEP or Section 504 Plan during any of their encounters with the HBSP (*p* = 0.240).

## Discussion

Although recommendations exist to support the developmental progress of pediatric patients with cardiac diagnoses (13), currently there are no guidelines available to medical teams that detail the school support needs while patients are hospitalized, during reintegration, or upon return to school, which may leave patients vulnerable to being under- or unserved and impact adulthood (6, 8, 27). Our sample of 29 pediatric cardiac patients had demonstrated a high use of school supports in the form of IEPs (58.62%) and Section 504 Plans (17.24%) by the end of the study period, compared with IEP (15%) (28) and Section 504 Plan rates (1%) in healthy peers (29). This is consistent with the literature highlighting the increased educational support necessary for pediatric patients with cardiac diagnoses (6, 30). Although this is a medically dissimilar population in terms of pathophysiology and specific medical interventions in terms of education and school service needs, there is high alignment due to significant neurocognitive and developmental impacts (12). Data capture did not include the IEP eligibility for these patients. The Individuals with Disabilities Education Improvement Act (IDEIA) (19) has 14 disability categories under which a patient may be found eligible, and based on what is known about the academic and medical needs of these patients, they may qualify for a variety of categories (e.g., other health impairments, multiple disabilities) (20). Database limitations impacted our ability to report what percentage of preexisting IEPs and Section 504 Plans were obtained with HBSP assistance in the years prior to the study period, and there were 24 patients who received a recommendation from the HBSP for new school support. Three of 8 patients received an IEP following a recommendation, and 1 of 13 patients received a Section 504 Plan following a recommendation during this period. HBSPs have significant variability, and they are designed to promote the access of pediatric patients to educational experts and specific reintegration planning between the medical team and the school (14, 15), which was achieved in this small cohort. To the authors' knowledge, this is one of the first studies examining the role of an HBSP for pediatric patients with complex cardiac diagnoses.

Despite our sample size limitations, high use of the HBSP was seen within this cohort, including the total number of encounters across two academic years; 68.97% of patients had multiple encounters in a single year, and 44.83% of patients were seen in both years. This level of patient engagement with the HBSP highlights the relative academic needs of this cohort. In addition, the HBSP communicated the changing academic needs of patients in multiple additional ways, including submitting to the school of record requests for classroom placement changes via medical homebound due to medical limitations impacting the schooling of patients (*N* = 27 times) and medical reports with recommendations impacting day-to-day school care and experience (*N* = 10 times). The HBSP also provided certificates of incapacity (*N* = 7 times), allowing for excused absenteeism without negative repercussions for these patients. While working with the medical team, the HBSP teachers were involved in decision-making regarding school recommendations. They communicated between school and medical teams, helping to translate medical needs into educational support (14, 31). Schools do perform their own eligibility determination tests and medical teams can support this process, for example, by working with an HBSP through information sharing that is pertinent to the school evaluation. Without the presence of the HBSP, the patients in this study may have had to wait longer for support initiation or may have had poorly aligned educational recommendations conveyed by the medical team alone.

Given the average length of stay of 24.35 days, which is equivalent to approximately 3 weeks of school in an academic year, ongoing instruction is important for maintaining the academic progress of these patients. To this end, the HBSP provided services directly to patients in the form of direct instruction, with patients averaging 9.83 sessions per hospitalization, amounting to almost two weeks of school time. The HBSP also provided school consultations to caretakers to promote family self-advocacy, with families receiving an average of 13.13 consultations during hospitalization. Again, this is reflective of a high utilization of HBSP services by the cohort.

The present study did not find any significant relationships between demographic factors (i.e., sex, race, ethnicity) or school level with academic services (i.e., IEPs, 504 Plans) for this cohort, suggesting no differences in receiving academic support recommendations. Information about how the type of school a patient attends impacts their access to educational support was limited (*N* = 2) in the private school setting. Given the high national rates of service utilization in pediatric cardiac patients (30), patients in private schools may be under- or unserved, as the IDEIA applies to public institutions (20) with more limited individualized service plans or access to services via the local public school for this population (20).

In this sample, no young children were present as the HBSP grant from the state DoE supported any child eligible for kindergarten (age 5 years) through 12th grade. Given what is known about the developmental and cognitive impact of these cardiac disorders and the intensity of treatment in early life, for those with congenital or acquired cardiac conditions specifically (1–4, 9–11), it would seem more likely that this group of patients would benefit from early evaluation and service initiation.

### Limitations

To the authors' knowledge, this is the first study to look at the role of HBSPs in supporting access to school services and accommodations for pediatric patients with cardiac diagnoses, and the limitations to consider when interpreting the findings include our small sample size, which restricted the analysis. Emerging patterns in this cohort may not apply to the larger population of children with complex congenital heart disease. Information about the sample population from the pre-2021–2022 academic year was not available, which limits our understanding of the role that the HBSP may have played for this group of patients in previous years. Additional information about the nature of the school supports in the form of specific recommendations made by the HBSP or as designed within the active IEP or Section 504 Plan was not available during this analysis, and only a preexisting IEP/Section 504 Plan or the implementation after the HBSP recommendation was available.

### Future directions

There are many study areas to expand on what is already known about the academic support for this population, including how to utilize HBSPs in promoting positive long-term outcomes for pediatric patients living with congenital or acquired cardiac conditions or for those in the postcardiac transplant period. The current literature has shown that educational outcomes like high school graduation and pursuit of secondary education are poor in this population (27). A key area for further study is to understand the risk factors for patient non-graduation related to medical and associated developmental and/or neurocognitive differences (32). Furthermore, finding methods to target and improve graduation rates or enrollment in vocational training for patients not eligible for diploma should be explored. A better understanding of patient school placement [e.g., general education or special education (18, 19)] and school-based services and interventions (e.g., reading intervention) may be important in developing a greater understanding of this area.

It would also be of interest to follow up those patients who received recommendations for a Section 504 Plan or who were served through a Section 504 Plan alone longitudinally to track whether further interventions or support are needed (e.g., IEP) later in their academic careers and whether these interventions affect graduation and/or post-high school education rates after the completion of high school (33). While standardization of support services in this pediatric population has been suggested, the development of guidelines that account for diverse education settings (e.g., private school, virtual school, etc.) and individual needs of patients to support reintegration would also be an area for further investigation (34). In addition, the 3-to-5-year-old group of patients who were not served by this HBSP due to funding limitations may benefit from school enrollment and service acquisition support in the future. Finally, in a relative value unit (RVU)-based medical practice where care coordination services such as those provided by this HBSP cannot be billed, having a support team that can interface with schools, medical teams, and families may be a cost-saving initiative to allow the medical team to generate RVUs while ensuring that quality of care is maintained.

### Summary

Pediatric patients with complex cardiac diagnoses have high developmental, cognitive, and academic/learning needs that may benefit from the advocacy, expertise, and support of specialized teachers in HBSPs during hospitalization and reintegration into school after discharge. HBSPs may serve as a bridge between medical experts, educational experts, and families working to build appropriate support and services for this vulnerable pediatric population.

## Data Availability

The raw data supporting the conclusions of this article will be made available by the authors without undue reservation.
